# Determination of chemical composition and metabolizable energy of chickpea, faba bean, field pea, lentil and lupin compared to soybean meal for broiler chickens

**DOI:** 10.1016/j.psj.2025.106286

**Published:** 2025-12-13

**Authors:** O. Hamungalu, M.R. Abdollahi, P.C.H. Morel, S. Liu, T.J. Wester

**Affiliations:** a School of Agriculture and Environment, Massey University, Palmerston North 4442, New Zealand; b Poultry Research Foundation, The University of Sydney, Camden NSW 2570, Australia

**Keywords:** Broiler, Grain legume, AME, AMEn, Chemical composition

## Abstract

The chemical composition, apparent metabolizable energy (**AME**) and nitrogen-corrected apparent metabolizable energy (**AMEn**) of lupin, faba bean, field pea, lentil, chickpea and soybean meal were determined for growing broilers. Grain legumes were incorporated into experimental diets either raw or after being steam-conditioned at 80°C for 30 seconds. Assay diets were developed by replacing (w/w) 300 g/kg of formulated basal diet (maize-soybean meal) with one of the legumes (raw or heat-treated) or soybean meal (test sample). Each assay diet was randomly allocated to 4 replicates (8 birds per cage) and fed in mash form for 7 d (d 14 – 21 post-hatch). Feed intake and total excreta were measured in the last 4 days of the trial. Among the grain legumes, starch content was lowest in lupin (4.3 g/kg) and highest in field pea (425 g/kg). Apart from lupin, grain legumes were greater in starch than soybean meal. Crude fat of soybean meal (13.5 g/kg) was lower than that of faba bean (14.1 g/kg), field pea (18.6 g/kg), lupin (47.2 g/kg) and chickpea (58.3 g/kg), but greater than lentil (10.9 g/kg). The gross energy of grain legumes ranged from 4,275 Kcal/kg (field pea) to 4,681 kcal/kg (lupin), and that of soybean meal was 4,514 Kcal/kg. Heating of grain legumes had no effect on AME and AMEn, except for lupin, where it increased by 23.9 and 23.5 %, respectively. The AME values among the raw legumes, were highest in lentil (2,438 Kcal/kg) and lowest in lupin (1,595 Kcal/kg), with the intermediate values for chickpea (2,206 Kcal/kg), faba bean (1,923 Kcal/kg) and pea (1,970 Kcal/kg). The AME of raw legumes were lower (*P<* 0.05) than that of soybean meal (2,673 Kcal/kg), but N-corrected AME of lentil was not different to that of soybean meal. The current results showed that steam conditioning at 80°C was not effective to improve energy availability of grain legumes, except for lupin. Soybean meal is superior to grain legumes in terms of energy availability for broiler feeding.

## Introduction

Dietary energy is an important component of broiler diet that influences bird performance and profitability (Farrell, 1999; Khalil et al., 2020). On average, 60 % of total broiler production cost is associated with feed cost (Kong and Adeola, 2014; Noblet, et al., 2022) and energy constitutes 70 % of that feed cost (Noblet et al., 2022). The energy in diets is largely supplied by cereal and plant-based protein sources like soybean meal (60 % and 30 %, respectively) (Khalil et al., 2023).

Although soybean meal (**SBM**) is widely used in broiler diets, many countries do not produce enough to meet local demand. Wide fluctuations in price of imported SBM create uncertainty for broiler producers and impacts sustainability (Nalle et al., 2012). Therefore, use of locally available ingredients might help reduce over-dependence on SBM, provide flexibility in diet formulation and reduce carbon footprint (Diaz et al., 2006; Kaczmarek et al., 2014; Nalle et al., 2012). Grain legumes, a potential alternative, can be grown in regions with climate conditions that are not ideal for soybean growing. For instance, faba bean, chickpea, lentil, field pea and lupin are more adaptive to temperate regions (Castell et al., 1996; Hamblin, 1987; Sipas et al., 1997). Globally, production of chickpea, lentil and lupin in 2023 were estimated at 16.5, 7.1 and 1.9 million tons, respectively, with Australia ranking first in lupin production (1.3 million tons) (FAOSTAT, 2025). Legumes fix nitrogen (**N**) into soil, hence reducing amount of N fertilizer added to the soil for crop growth (Adamidou et al., 2011; Kaczmarek et al., 2014).

Published nutritional values of grain legume are variable (Kaczmarek et al., 2014; Nalle et al., 2011, 2012) and attributed mainly to cultivar effects (Hughes et al., 1998; Koivunen et al., 2016; Nalle et al., 2010b, 2012), climate and geographical location (Hughes et al., 1998). Koivunen et al. (2016) reported apparent metabolizable energy (**AME**) range of 2,914 – 3,296, 2,842 – 2,962, and 1,672 Kcal/kg for field pea, faba bean and lupin fed to 28 d old broilers, respectively. Nalle et al. (2011) reported N-corrected AME (**AMEn**) values ranging from 1,278 to 1,476 Kcal/kg of three lupin cultivars fed to 32 d old broilers.

Furthermore, continual improvement in grain legume breeding (Cargo-Froom et al., 2023) necessitates provision of updated nutritional values to improve diet formulation for better performance of birds. Therefore, the objective of this study was to characterise chemical composition and determine AME and AMEn for lupin, lentil, chickpea, field pea and faba bean grown in Australia compared to SBM for growing broiler chickens. Also, to examine the influence of steam conditioning of grain legumes on energy availability.

## Materials and methods

The experiment was conducted according to the New Zealand Code of Ethical Conduct for the use of live animals for research, testing and teaching and approved by the Massey University Animal Ethics Committee.

### Ingredients

Grain legumes (faba bean, chickpea, field pea, lentil and lupin) were sourced from a commercial supplier in Australia and ground through a 2.0 mm sieve. Soybean meal (solvent extracted) and maize were sourced from a commercial supplier. A portion of each legume was steam conditioned in the pelleting chamber (Model Orbit 15; Richard Sizer Ltd., Kingston-upon-Hull, UK) at 80°C for 30 seconds and then air dried for five days.

### Diets

The AME and AMEn for grain legumes (5 raw and 5 heat-treated samples) and SBM were determined by the difference method. In this method, maize-SBM basal diet was formulated (Table 1) and used to develop test diets by replacing (w/w) 300 g/kg of the basal diet with one of the legumes (raw or heat-treated) or SBM.

**Table 1:** Composition of the basal diet (g/kg, as fed basis).

Ingredient	Inclusion, g/kg
Maize	604.4
Soybean meal, 460 g/kg	338.1
Soybean oil	14.2
Dicalcium phosphate	15.8
Limestone	10.4
L-Lysine	3.7
DL-Methionine	3.1
L-Threonine	2.0
L-Valine	0.7
Sodium Chloride	1.0
Sodium bicarbonate	3.9
Trace mineral premix^1^	1.0
Vitamin premix^1^	1.0
Choline chloride 60 %	0.7

1 Supplied per kg of diet: antioxidant, 100 mg; biotin, 0.2 mg; calcium pantothenate, 12.8 mg; vitamin D3 (cholecalciferol), 2400 IU; cyanocobalamin, 0.017 mg; folic acid, 5.2 mg; menadione, 4 mg; niacin, 35 mg; pyridoxine, 10 mg; vitamin A (trans-retinol), 11100 IU; riboflavin, 12 mg; thiamine, 3.0 mg; vitamin E (dl-α-tocopheryl acetate), 60 IU; choline chloride, 638 mg; Co, 0.3 mg; Cu, 3.0 mg; Fe, 25 mg; I, 1 mg; Mn, 125 mg; Mo, 0.5 mg; Se, 200 µg; Zn, 60 mg.

### Birds and housing

Day-old male broilers (Ross 308) obtained from a commercial hatchery were raised in floor pens (length 1.6 m x width 0.8 m) and moved to cages (length 0.6 m x width 0.6 m) on d-14. On d-14, a total of 384 birds were randomly allocated to 48 cages, with 8 birds per cage and 4 replicated cages per dietary treatment. Before the introduction of test diets on d-14, birds were fed a standard starter diet (230 g/kg crude protein and 3,010 Kcal/kg AMEn). The floor pens and cages were housed in an environmentally controlled room supplying 20 h of fluorescent illumination per day. During the first week, temperature was maintained at 31°C and then gradually reduced to 23°C by the third week of age. Feed and water were supplied *ad libitum* throughout the trial period.

### Determination of apparent metabolizable energy

A total excreta collection procedure (Hill and Anderson, 1958) was used to determine AME. Experimental diets were fed for 7 days from 14 to 21 d post-hatch, the first 3 d as adaptation period. During the last 4 d, feed intake (**FI**) was recorded, and excreta collected and weighed daily. The daily excreta were pooled by cage, frozen, mixed in a blender and representative sub-samples obtained and freeze-dried (Model 0610, Cudon Engineering, Blenheim, New Zealand). Diets and excreta samples were ground to pass through a 0.5 mm sieve and stored in airtight plastic containers at 4°C pending analysis of dry matter (**DM**), gross energy (**GE**) and N.

### Chemical analysis

The DM of ingredients, diets and excreta was determined using standard procedures (Methods 930.15 and 925.10; AOAC, 2016). Nitrogen was determined by combustion (Method 968.06; AOAC, 2016) using a carbon nanosphere-200 carbon, N and sulphur autoanalyser (LECO Corporation, St. Joseph, MI). Crude fat was determined by Soxtec extraction procedure (Method 2003.06; AOAC, 2016). The GE was determined by a adiabatic bomb calorimetry (Gallenkamp Autobomb, Weiss Gallenkamp Ltd, Loughborough, UK) standardised with benzoic acid. Starch content was determined using a Megazyme kit (Method 996.11; AOAC, 2016) based on thermostable α-amylase and amyloglucosidase. Neutral detergent fibre (**NDF**), acid detergent fibre (**ADF**) and lignin were measured (Method 2002.04; AOAC, 2016) using Tecator Fibertec™ (FOSS Analytical AB, Höganäs, Sweden). Soluble dietary fibre (**SDF**), insoluble dietary fibre (**IDF**) and total dietary fibre (**TDF**) were measured using Megazyme total dietary fibre assay kit (Method 991.43; AOAC, 2016).

Ash was determined by standard procedures (Method 942.05; AOAC, 2016) using a muffle furnace at 550°C for 16 hours. The content of minerals was measured by inductively coupled plasma-optical emission spectroscopy (ICP-OES) using a Thermo Jarrell Ash IRIS instrument (Thermo Jarrell Ash Corporation, Franklin, MA).

Resistant starch for grain legumes was determined using a resistant starch assay kit (Megazyme International Ireland Ltd., Wicklow, Ireland; AOAC Method 2002.02 and AACC Method 32-40.01) based on pancreatic α-amylase and amyloglucosidase (Englyst et al., 1994; McCleary and Monaghan, 2002). Samples were incubated in a shaking water bath with enzymes for 16 h at 37°C, during which time non-resistant starch was solubilised and hydrolysed to d-glucose by the combined action of the two enzymes.

### Calculations

All data were expressed on a DM basis and AME was determined using the following difference method formulae (Khalil et al., 2023):

$$\text{AM}E_{\text{Diet}}\left(\text{Kcal}/\text{kg}\right)\;=\;\left[\left(\text{FI}\;\times \;GE_{\text{Diet}}\right)\;-\;\left(\text{Excreta}\;\text{output}\;\times GE_{\text{Excreta}}\right)\right]\;/\;\text{FI}$$

$$\text{AM}E_{\text{Legume}/\text{SBM}}\left(\text{Kcal}/\text{kg}\right)\;=\;\left[\text{AME}\;\text{of}\;\text{test}\;\text{ingredient}\;\text{diet}\;-\;\left(\text{AME}\;\text{of}\;\text{basal}\;\text{diet}\;\times \;0.70\right)\right]\;/\;0.30$$

Nitrogen retention, as a percentage of intake, was determined as follows:

$$N_{\text{retention}\;\left(\%\right)}\;=\;100\;\times \;\left[\left(\left(\text{FI}\;\times \;N_{\text{diet}\;}\right)\;-\;\left(\text{Excreta}\;\text{output}\;\times \;N_{\text{excreta}}\right)\right)\;/\;\left(\text{FI}\;\times \;N_{\text{diet}}\right)\right]$$

Determination of AMEn was by correcting for zero N retention by multiplication with 8.73 Kcal per g N retained in the body as described by Titus et al. (1959).

### Statistical analysis

The data were analysed using the General Linear Models procedure of SAS (version 9.4; 2015. SAS Institute Inc., Cary, NC). One-way ANOVA and orthogonal contrasts (raw vs heat-treated grain legume; SBM vs raw grain legume; SBM vs heat-treated grain legume) were performed. Cage served as the experimental unit. Differences were considered significant at *P <* 0.05.

## Results

Among grain legumes, starch content was lowest in lupin and highest in field pea (Table 2). Apart from lupin, grain legumes were greater in starch than SBM. Crude fat in grain legumes was greater than in SBM, except for lentil that had low fat content. The TDF was greatest in lupin followed by faba bean and lowest in lentil, among grain legumes. The GE for grain legumes (4,275 – 4,681 Kcal/kg) was comparable to SBM (4,514 Kcal/kg).

**Table 2:** Proximate, carbohydrate, mineral and resistant starch composition of test grain legumes and soybean meal (g/kg, DM).

Item	Chickpea	Faba	Lentil	Lupin	Pea	Soybean meal
Dry matter	926	919	914	933	916	891
Nitrogen	35.6	42.7	44.8	45.6	30.4	73.8
Ash	29.2	30.5	21.9	27.9	28.4	69.6
Fat	58.3	14.1	10.9	47.2	18.6	13.5
Starch	419	380	387	4.3	425	34.8
ADF	18.4	94.7	37.2	248	71.0	49.4
NDF	54.0	148	73.3	325	115	119
Lignin	5.4	5.4	7.7	19.3	6.6	7.9
IDF	108	201	121	536	201	192
SDF	22.7	19.6	7.7	52.5	16.4	21.3
TDF	131	221	129	588	217	213
GE (kcal/kg)	4,538	4,347	4,323	4,681	4,275	4,514
Calcium	1.22	1.16	0.76	3.43	0.68	3.93
Phosphorus	3.78	4.35	2.85	2.79	3.60	7.52
Magnesium	1.50	1.36	1.04	1.98	1.18	3.59
Potassium	11.3	11.5	8.97	7.72	12.0	26.9
Sodium	0.055	0.157	0.055	0.225	0.152	0.134
Iron	0.045	0.062	0.059	0.038	0.073	0.127
Copper	0.004	0.008	0.008	0.003	0.004	0.016
Manganese	0.027	0.040	0.013	0.101	0.013	0.058
Zinc	0.033	0.044	0.028	0.029	0.025	0.052
Resistant starch^1^	338.8	176.2	322.6	0.00	189.9	-

1 As received basis. GE = gross energy, NDF = neutral detergent fiber, ADF = acid detergent fiber, IDF = insoluble dietary fiber, SDF = soluble dietary fiber, TDF = total dietary fiber.

Calcium and phosphorus content in SBM was greater than that of grain legumes. Among grain legumes, calcium was lowest (0.68 g/kg) and highest (3.43 g/kg) in field pea and lupin, respectively. While for phosphorus, lupin was lowest and faba bean the highest. Resistant starch in grain legumes was highest in chickpea and lowest (non-detected) in lupin.

The AME was highest in lentil (2,438 Kcal/kg; Table 3) followed by chickpea (2,206 Kcal/kg) and lowest in lupin (1,595 Kcal/kg). The AME in faba bean and field pea were 1,923 and 1,970 Kcal/kg, respectively. The AMEn showed a pattern similar to that of AME.

**Table 3:** Apparent metabolizable energy (AME; Kcal/kg, DM) and N-corrected apparent metabolizable energy (AMEn; Kcal/kg, DM) of raw and heat-treated grain legumes.

Item	Chickpea		Faba		Lentil		Lupin		Pea		SEM	P value^1^	P value^2^				
													Raw vs. heat treated				
	Raw	HT	Raw	HT	Raw	HT	Raw	HT	Raw	HT			chickpea	Faba	Lentil	Lupin	Pea
AME	2,206	2,237	1,923	1,906	2,438	2,539	1,595	1,976	1,970	1,948	54.82	0.001	0.693	0.831	0.196	0.001	0.770
AMEn	2,108	2,128	1,777	1,784	2,318	2,405	1,465	1,809	1,857	1,826	51.44	0.001	0.779	0.928	0.231	0.001	0.672

SEM = standard error of the mean, HT = steam conditioned for 30 s at 80°C.

1 P value for ANOVA is significantly different at *P<* 0.05.

2 P value for orthogonal contrast indicates significance (*P<* 0.05) of raw vs heat-treated grain legumes.

No significant (*P >* 0.05; Table 3) effect of heating was observed on AME and AMEn for faba bean, chickpea, lentil and field pea. However, heating significantly (*P <* 0.05) increased AME and AMEn for lupin (23.9 and 23.5 %, respectively).

The AME of SBM was greater (*P <* 0.05; Table 4) than that of raw grain legumes, however, AMEn of lentil was not different from SBM (*P >* 0.05). Also, only AMEn:GE of lentil was similar to SBM (*P >* 0.05; Table 4). For heat-treated grain legumes, only AME and AMEn of lentil were not lower than that of SBM (*P >* 0.05; Table 5).

**Table 4:** Apparent metabolizable energy (AME; kcal/kg, DM), N-corrected apparent metabolizable energy (AMEn; Kcal/kg, DM) and ratio of AMEn to gross energy (AMEn:GE; Kcal/kg) of raw grain legumes compared to soybean meal.

Item	CKP	Faba	Lentil	Lupin	Pea	SBM	SEM	P value^1^	P value^2^				
									Raw Legume vs. SBM				
									CKP	Faba	Lentil	Lupin	Pea
AME	2,206	1,923	2,438	1,595	1,970	2,673	54.82	0.001	0.001	0.001	0.003	0.001	0.001
AMEn	2,108	1,777	2,318	1,465	1,857	2,402	51.44	0.001	0.001	0.001	0.245	0.001	0.001
AMEn:GE	0.465	0.409	0.536	0.313	0.434	0.532	0.011	0.001	0.001	0.001	0.807	0.001	0.001

SEM = Standard error, CKP = Chickpea, SBM = soybean meal.

1 P value for ANOVA is significantly different at *P<* 0.05.

2 P value for orthogonal contrast indicates significance of raw vs SBM.

**Table 5:** Apparent metabolizable energy (AME; Kcal/kg, DM) and N-corrected apparent metabolizable energy (AMEn; Kcal//kg, DM) of heat-treated grain legumes compared to soybean meal.

Item	CKP	Faba	Lentil	Lupin	Pea	SBM	SEM	P value^1^	P value^2^				
									Heat-treated Legume vs. SBM				
									CKP	Faba	Lentil	Lupin	Pea
AME	2,237	1,906	2,539	1,976	1,948	2,673	54.82	0.001	0.001	0.001	0.086	0.001	0.001
AMEn	2,128	1,784	2,405	1,809	1,826	2,402	51.44	0.001	0.001	0.001	0.971	0.001	0.001

SEM = standard error, CKP = Chickpea, SBM = soybean meal.

1 P value for ANOVA is significantly different at *P<* 0.05.

2 P value for orthogonal contrast indicates significance of raw vs SBM.

## Discussion

Starch is the largest contributor of dietary energy in broiler diets (Wang et al., 2003). In the present study, starch in field pea, chickpea and faba bean agreed with published literature (Adamidou et al., 2011; Nalle et al., 2010b). Starch content was lowest in lupin among the grain legumes. Knudsen (1997) reported 14 g/kg starch in lupin, while in Nalle et al. (2011) starch was not detected in lupin. The starch content of lentil was greater than the value (286 g/kg) reported by Woyengo et al. (2014), but lower than that of Hefnawy (2011), and Wang and Daun (2006). Wang and Daun (2006) observed variation in chemical composition of lentil cultivars and attributed this to genetic, agronomical and geographical factors.

Lupin was high in dietary fiber in the present study. Dietary fiber is largely composed of non-starch polysaccharides (**NSP**) and lignin (Knudsen, 1997). Nalle et al. (2011) reported total NSP in lupin of 432 to 496 g/kg. High NSP in lupin was also reported in other studies (Nalle et al., 2012; Perez-Maldonado et al., 1999; Ravindran et al., 2002).

Crude fat content in chickpea and lentil corresponded to values in published studies (Adamidou et al., 2011; Thacker et al., 2002; Viveros et al., 2001) and (Hefnawy, 2011; Wang and Daun, 2006), respectively. The content of crude fat in lupin (47.2 g/kg) was within the range reported by Brand et al. (2018), but lower than that of other studies (Koivunen et al., 2016; Nalle et al., 2012; Ravindran et al., 2002). While crude fat in field pea and faba bean agreed with data reported by Igbasan and Guenter (1996) and Adamidou et al. (2011), respectively.

In the present study, AME and AMEn of faba bean were lower than those reported by Nalle et al. (2010b). Nalle et al. (2010b) reported varied AME (2,102 – 2,859 Kcal/kg) of faba bean and attributed it to cultivar differences. Information on the cultivar of grain legumes used in the present study was not available. The AME of lupin was within values (1,524 – 1,701 kcal/kg) reported by Nalle et al. (2011), but lower than values (1,923 – 2,312 Kcal/kg) of Nalle et al. (2012). Low energy associated with lupin compared to other legumes and SBM is attributed mainly to low starch and high fiber content (Kaczmarek et al., 2014). Fiber is generally inversely related to energy availability in broiler feeding (Hughes et al., 1998; Knudsen, 1997). According to Hughes et al. (1998), the inclusion of NSP at 150 g/kg in a maize-based broiler diet reduced AME by 29 % (3,463 vs 2,460 Kcal/kg). They also observed improved AME for dehulled lupin since fiber is concentrated in the seed coat. Likewise, Nalle et al. (2010a) observed increased AMEn for dehulled lupin. Grain legumes in the present study were not dehulled.

Compared to SBM, energy utilization of grain legumes was poor. In the present study, AME of SBM was greater than that of raw grain legumes, however, AMEn of lentil was not different from SBM. This could be due to N-correction of high protein SBM (Abdollahi et al., 2021). Furthermore, although GE of grain legumes was comparable to SBM, only the AMEn:GE ratio of lentil was similar to that of SBM. The grain legumes in the present study were relatively low in trypsin inhibitor and phytic acid (Hamungalu et al. unpublished data), but high in resistant starch. Heating at 80C for 30 s had a modest influence on trypsin inhibitor and phytic acid, but except for chickpea and field pea (3.48 and 0.89 mg/g trypsin inhibitor activity, respectively), values in raw samples were already as low as or lower than processed SBM (0.66 mg/g trypsin inhibitor activity). The starch of grain legumes generally belongs to type C, the slowly digested category (Gunawardena et al., 2010). Sugimoto (1980) reported high resistance of field pea and faba bean starch to pancreatin attack. This is mostly due to high amylose to amylopectin ratio and large size of starch granules in grain legumes (Anguita et al., 2006). A large starch granule is associated with small surface area to volume ratio that limits amylase attachment and leads to reduced starch digestion (Tester et al., 2004). Also, starch high in amylopectin is generally more digestible (Karr-Lilienthal et al., 2005). Therefore, it can be speculated that better understanding of legume starch and cell walls could provide ways to improve energy availability for broiler feeding.

The benefit of heating grain legumes was only observed in lupin that had AME and AMEn increased by 23.9 and 23.5 %, respectively. This agreed to findings of Rutkowski et al. (2016), who reported increased AMEn by 11.6 % (2,276 vs 2,541 Kcal/kg) due to extrusion. They also reported improved apparent ileal fat and protein digestibility.

The AME and AMEn of heat-treated lentil were not different to that of SBM. While for field pea, there was a slight reduction in AME due to heating. This is contrary to the findings of Carre et al. (1991) involving steam pelleted (75-81°C) and re-ground pea. They reported increased starch digestibility and AMEn by 18.3 and 15.3 %, respectively. Skoch et al. (1981) observed a temperature difference of 6-10°C when temperature of a pelleted diet was compared to that of diet in the conditioning chamber. The observation was attributed to mechanical shear stress as feed is forced through the pellet die. Grain legumes in the present study were not pelleted, but only steam-conditioned, therefore, heating may not have been adequate to induce gelatinization, which facilitates starch digestion (Hugman et al., 2021). Heat disrupts cell walls and initiates gelatinization, which facilitates digestion of starch granules by alpha-amylase (Conan and Carre, 1989; Rutkowski et al., 2016). Lacassagne et al. (1988) attributed increased AMEn of a pelleted (66-71°C) diet to improved starch digestibility.

## Conclusions

This study showed that energy utilization in SBM is better than that in grain legumes despite having comparable GE. Therefore, supplemental energy sources, such as oil might be required to balance broiler diets that contain grain legumes. It was also shown that steam conditioning at 80°C was not effective in improving energy availability of grain legumes, except for lupin, suggesting variable effects of different processing techniques.

## CRediT authorship contribution statement

**O. Hamungalu:** Writing – review & editing, Writing – original draft, Investigation, Formal analysis, Data curation, Conceptualization. **M.R. Abdollahi:** Writing – review & editing, Supervision, Investigation, Funding acquisition, Formal analysis, Conceptualization. **P.C.H. Morel:** Writing – review & editing, Supervision, Formal analysis, Data curation. **S. Liu:** Writing – review & editing, Supervision, Conceptualization. **T.J. Wester:** Writing – review & editing, Supervision, Project administration, Investigation, Funding acquisition, Conceptualization.

## Disclosures

The authors declare the following financial interests/personal relationships which may be considered as potential competing interests:

Timothy J Wester, M. Reza Abdollahi, Obright Hamungalu, Sonia Liu reports financial support, article publishing charges, equipment, drugs, or supplies, and travel were provided by AgriFutures Australia. If there are other authors, they declare that they have no known competing financial interests or personal relationships that could have appeared to influence the work reported in this paper.

## References

[bib0001] Abdollahi M.R., Wiltafsky-Martin M.., Ravindran V. (2021). Application of apparent metabolizable energy versus nitrogen-corrected apparent metabolizable energy in poultry feed formulations: a continuing conundrum. Anim. Sci..

[bib0002] Adamidou S., Nengas I., Grigorakis K., Nikolopoulou D., Jauncey K. (2011). Chemical composition and antinutritional factors of field peas (Pisum sativum), chickpeas (Cicer arietinum), and faba beans (Vicia faba) as affected by extrusion preconditioning and drying temperatures. Cereal Chem..

[bib0003] Anguita M., Gasa J., Martín-Orúe S.M., Pérez J.F. (2006). Study of the effect of technological processes on starch hydrolysis, non-starch polysaccharides solubilization and physicochemical properties of different ingredients using a two-step in vitro system. Anim. Feed Sci. Technol..

[bib0004] AOAC International (2016).

[bib0005] Brand T., Engelbrecht J., Van der Merwe J., Hoffman L. (2018). Feed preference of grower ostriches consuming diets differing in Lupinus angustifolius inclusion levels. S. Afr. J. Anim. Sci..

[bib0006] Cargo-Froom C.L., Tansil F.., Columbus D.A., Marinangeli C.P.F., Kiarie E.G., Shoveller A.k. (2023). Determination of standardized ileal digestibility of crude protein and amino acids and digestible indispensable amino acid score of faba beans, lentils, and yellow peas fed to growing pigs. Can. J. Anim. Sci..

[bib0007] Carre B., Beaufils E., Melcion J.P. (1991). Evaluation of protein and starch digestibility and energy value of pelleted or unpelleted pea seeds from winter or spring cultivars in adult and young chickens. J. Agric. Food Chem..

[bib0008] Castell A., Guenter W., Igbasan F. (1996). Nutritive value of peas for nonruminant diets. Anim. Feed Sci. Technol..

[bib0011] Conan L., Carre B. (1989). Effect of autoclaving on metabolizable energy value of smooth pea seed (Pisum sativum) in growing chicks. Anim. Feed Sci. Technol..

[bib0012] Diaz D., Morlacchini M., Masoero F., Moschini M., Fusconi G., Piva G. (2006). Pea seeds (Pisum sativum), faba beans (Vicia faba var. minor) and lupin seeds (Lupinus albus var. multitalia) as protein sources in broiler diets: effect of extrusion on growth performance. Ital. J. Anim. Sci..

[bib0013] Englyst H.N., Quigley M..E., Hudson G.J. (1994). Determination of dietary fibre as non-starch polysaccharides with gas–liquid chromatographic, high-performance liquid chromatographic or spectrophotometric measurement of constituent sugars. Analyst.

[bib0014] FAOSTAT. 2025. Statistics Database of the Food and Agriculture Organization of the United Nations. Accessed May 2025. https://www.fao.org/faostat/en/#data.

[bib0015] Farrell D. (1999). In vivo and in vitro techniques for the assessment of the energy content of feed grains for poultry: a review. Aust. J. Agric. Res..

[bib0016] Gunawardena C.K., Zijlstra R..T., Beltranena E. (2010). Characterization of the nutritional value of air-classified protein and starch fractions of field pea and zero-tannin faba bean in grower pigs. J. Anim. Sci..

[bib0017] Hamblin J. (1987). Proc. 4th Australia Agron.

[bib0018] Hefnawy T. (2011). Effect of processing methods on nutritional composition and anti-nutritional factors in lentils (Lens culinaris). Ann. Agric. Sci..

[bib0019] Hill F., Anderson D. (1958). Comparison of metabolizable energy and productive energy determinations with growing chicks. J. Nutr..

[bib0020] Hughes R., Kocher A., Choct M. (1998). Proc. Aust. Poult. Sci. Sym.

[bib0021] Hugman J., Wang L.F., Beltranena E., Htoo J.K., Vasanthan T., Zijlstra R.T. (2021). Energy and amino acid digestibility of raw, steam-pelleted and extruded red lentil in growing pigs. Anim. Feed Sci. Technol..

[bib0022] Igbasan F., Guenter W. (1996). The evaluation and enhancement of the nutritive value of yellow-, green-and brown-seeded pea cultivars for unpelleted diets given to broiler chickens. Anim. Feed Sci. Technol..

[bib0023] Kaczmarek S., Kasprowicz-Potocka M., Hejdysz M., Mikuła R., Rutkowski A. (2014). The nutritional value of narrow-leafed lupin (Lupinus angustifolius) for broilers. J. Anim. Feed Sci..

[bib0024] Karr-Lilienthal L., Kadzere C., Grieshop C., Fahey Jr G. (2005). Chemical and nutritional properties of soybean carbohydrates as related to nonruminants: a review. Livest. Prod. Sci..

[bib0025] Khalil M., Abdollahi M.R., Zaefarian F., Ravindran V. (2020). Measurement of ileal endogenous energy losses and true ileal digestible energy of cereal grains for broiler chickens. Poult. Sci..

[bib0026] Khalil M.M., Abdollahi M..R., Zaefarian F., Chrystal P.V., Ravindran V. (2023). Broiler age influences the apparent metabolizable energy of soybean meal and canola meal. Anim.

[bib0027] Knudsen K.E.B. (1997). Carbohydrate and lignin contents of plant materials used in animal feeding. Anim. Feed Sci. Technol..

[bib0028] Koivunen E., Partanen K., Perttilä S., Palander S., Tuunainen P., Valaja J. (2016). Digestibility and energy value of pea (Pisum sativum L.), faba bean (Vicia faba L.) and blue lupin (narrow-leaf) (Lupinus angustifolius) seeds in broilers. Anim. Feed Sci. Technol..

[bib0029] Kong C., Adeola O. (2014). Evaluation of amino acid and energy utilization in feedstuff for swine and poultry diets. Asian-Austral. J. Anim. Sci..

[bib0030] Lacassagne L., Francesch M., Carré B., Melcion J. (1988). Utilization of tannin-containing and tannin-free faba beans (Vicia faba) by young chicks: effects of pelleting feeds on energy, protein and starch digestibility. Anim. Feed Sci. Technol..

[bib0031] McCleary B.V., Monaghan D.A. (2002). Measurement of resistant starch. J. Assoc. Off. Anal. Chem..

[bib0032] Nalle C.L., Ravindran V.., Ravindran G. (2011). Nutritional value of narrow-leafed lupin (Lupinus angustifolius) for broilers. Br. Poult. Sci..

[bib0033] Nalle C.L., Ravindran G.., Ravindran V. (2010). Influence of dehulling on the apparent metabolizable energy and ileal amino acid digestibility of grain legumes for broilers. J. Sci. Food Agric..

[bib0034] Nalle C.L., Ravindran V.., Ravindran G. (2010). Nutritional value of faba beans (Vicia faba L.) for broilers: apparent metabolizable energy, ileal amino acid digestibility and production performance. Anim. Feed Sci. Technol..

[bib0035] Nalle C.L., Ravindran V.., Ravindran G. (2012). Nutritional value of white lupins (Lupinus albus) for broilers: apparent metabolizable energy, apparent ileal amino acid digestibility and production performance. Anim. Sci..

[bib0036] Noblet J., Wu S.B., Choct M. (2022). Methodologies for energy evaluation of pig and poultry feeds: a review. Anim. Nutr..

[bib0037] Perez-Maldonado R., Mannion P., Farrell D. (1999). Optimum inclusion of field peas, faba beans, chick peas and sweet lupins in poultry diets. I. Chemical composition and layer experiments. Br. Poult. Sci..

[bib0038] Ravindran V., Tabe L.M., Molvig L., Higgins T.J.V., Bryden W.L. (2002). Nutritional evaluation of transgenic high-methionine lupins (Lupinus angustifolius L) with broiler chickens. J. Sci. Food Agric..

[bib0039] Rutkowski A., Kaczmarek S.A., Hejdysz M., Jamroz D. (2016). Effect of extrusion on nutrients digestibility, metabolizable energy and nutritional value of yellow lupin seeds for broiler chickens. Ann. Anim. Sci..

[bib0041] Sipas S., Mackintosh J.B., Petterson D.S. (1997).

[bib0042] Skoch E., Behnke K., Deyoe C., Binder S. (1981). The effect of steam-conditioning rate on the pelleting process. Anim. Feed Sci. Technol..

[bib0043] Sugimoto Y. (1980). Scanning electron microscopic observation of starch granules. J. Jpn. Soc. Starch Sci..

[bib0044] Tester R.F., Karkalas J.., Qi X. (2004). Starch structure and digestibility enzyme-substrate relationship. Worlds Poult. Sci. J..

[bib0045] Thacker P.A., Qiao S.., Racz V.J. (2002). A comparison of the nutrient digestibility of Desi and Kabuli chickpeas fed to swine. J. Sci. Food Agric..

[bib0046] Titus H.W., Mehring Jr A..L., Johnson Jr D., Nesbitt L.L., Tomas T. (1959). An evaluation of MCF (Micro-Cel-Fat), a new type of fat product. Poult. Sci..

[bib0047] Viveros A., Brenes A., Elices R., Arija I., Canales R. (2001). Nutritional value of raw and autoclaved kabuli and desi chickpeas (Cicer arietinum L.) for growing chickens. Poult. Sci..

[bib0048] Wang N., Daun J. (2006). Effects of variety and crude protein content on nutrients and anti-nutrients in lentils. Food Chem..

[bib0049] Wang T.L., Domoney C.., Hedley C.L., Casey R., Grusak M.A. (2003). Can we improve the nutritional quality of legume seeds?. Plant Physiol..

[bib0050] Woyengo T., Jha R., Beltranena E., Pharazyn A., Zijlstra R. (2014). Nutrient digestibility of lentil and regular-and low-oligosaccharide, micronized full-fat soybean fed to grower pigs. J. Anim. Sci..

